# A free-access online key to identify Amazonian ferns

**DOI:** 10.3897/phytokeys.78.11370

**Published:** 2017-03-22

**Authors:** Gabriela Zuquim, Hanna Tuomisto, Jefferson Prado

**Affiliations:** 1 Department of Biology, University of Turku, FI-20014, Finland; 2 Brazilian National Program for Biodiversity Research (PPBio), Av. André Araújo, 2.936, 69060-001, Manaus, AM, Brazil; 3 Instituto de Botânica, Herbário SP, Av. Miguel Estéfano, 3687, 04301-902, São Paulo, SP, Brazil

**Keywords:** Amazonia, Ferns, Identification key, Indicator species, Lucid3, Free-access key, Online identification tools, Pteridophyte, Tropical forests

## Abstract

There is urgent need for more data on species distributions in order to improve conservation planning. A crucial but challenging aspect of producing high-quality data is the correct identification of organisms. Traditional printed floras and dichotomous keys are difficult to use for someone not familiar with the technical jargon. In poorly known areas, such as Amazonia, they also become quickly outdated as new species are described or ranges extended. Recently, online tools have allowed developing dynamic, interactive, and accessible keys that make species identification possible for a broader public. In order to facilitate identifying plants collected in field inventories, we developed an internet-based free-access tool to identify Amazonian fern species. We focused on ferns, because they are easy to collect and their edaphic affinities are relatively well known, so they can be used as an indicator group for habitat mapping. Our key includes 302 terrestrial and aquatic entities mainly from lowland Amazonian forests. It is a free-access key, so the user can freely choose which morphological features to use and in which order to assess them. All taxa are richly illustrated, so specimens can be identified by a combination of character choices, visual comparison, and written descriptions. The identification tool was developed in Lucid 3.5 software and it is available at http://keyserver.lucidcentral.org:8080/sandbox/keys.jsp.

## Introduction

“*Science is a sequence of generating new ideas, detailed explorations, incorporation of the results into a toolbox for understanding data, and turning them into useful knowledge.*” Peterson et al. 2010

The first species identification key was published more than 330 years ago (Griffing 2011). It was a dichotomous key, which means that each question about the morphological characters of the specimen has two alternative answers, and the order in which the characters are assessed is pre-determined by the designer of the key (Hagedorn et al. 2010). A multientry identification system based on punch cards was developed already in the 1960’s (Hansen and Rahn 1969). Nevertheless, until the bioinformatics revolution in the last 20 years (Peterson et al. 2010), most people wishing to make species identifications were forced to either use dichotomous single-entry keys or solicit personal assistance from experts (Hardisty et al. 2013). For most parts of the world, compiled identification guides have not been available, so the identification keys, species names, and species descriptions have been scattered in the scientific literature (Polaszek 2005). This has caused taxonomic information to only be accessible for a restricted group of specialists at universities and research institutions with extensive library facilities.

The rise of the Internet has been a significant development for systematics (Farr 2006). First, the Internet makes it possible to deliver taxonomic information to end-users directly, immediately and globally, mitigating important aspects of the taxonomic impediment (Ebach et al. 2011). Second, publishing information online makes rapid and frequent updating of taxonomic works feasible; no expensive printing of a new edition is needed. This flexibility is much needed, given that ca. 25,000 new scientific names for organisms are proposed every year (Knapp et al. 2007). Third, the Internet facilitates the development of identification formats other than the dichotomous single-access keys based on verbal descriptions. Currently, three kinds of keys are found online: single-access, free-access, and hybrid (Hagedorn et al. 2010). In free-access keys, it is up to the user to decide which characters and in which sequence to use. Often, guidance in character selection is offered, there is tolerance for errors, and the character choices can be changed at any time (Dallwitz et al. 2000). In contrast, single-access keys provide a single path for each result, errors are not tolerated, and if a diagnostic character is missing, identification cannot be continued beyond the point in the key where that character is used (Hagedorn et al. 2010). For these reasons, free-access keys are more flexible and have several advantages over single-access keys.

With the rapid progress in online approaches, taxonomy has become more accessible and integrated to the society. Newly developed software like Lucid (http://www.lucidcentral.com/), FRIDA (Martellos 2010), Intkey (Dallwitz et al. 1993) and Xper (Ung et al. 2010) have made it technically feasible for anyone to produce web-based identification keys that are dynamic, interactive, and easily accessible. Moreover, online keys can make almost unlimited use of colorful photographs and other illustrations, which are constrained by printing costs in paper keys, guides and floras (Brach and Song 2006). Inclusion of illustrations in identification keys is very important. Most people find it more intuitive to identify a specimen by comparing it with illustrations of diagnostic characteristics than by comparing it with verbal descriptions of those characteristics. Therefore, enabling visual assessment can greatly enhance the usability of taxonomic works, and can also be expected to result in more reliable identifications, especially when done by non-experts (Martellos and Nimis 2015). In addition, online tools can take advantage of additional information by incorporating dynamic objects (such as scalable species distribution maps), associated files (such as PDF versions of taxonomic publications) and links to information published elsewhere in the Internet. All of this is unattainable in printed keys.

In the last 20 years, a wide range of tools has been developed to address different societal demands on species identification. Crowd source identification (http://www.inaturalist.org/) and taxonomic platforms (http://scratchpads.eu/), mobile apps (http://bien.nceas.ucsb.edu/bien/tools/plant-o-matic/, http://leafsnap.com/), and even games (Seidman et al. 2016) are contributing to a quick delivery of taxonomic information to a global audience. Some keys have more applied purposes, like identifying pests and crop diseases (http://www.idtools.org/id/citrus/diseases/key.php; http://idtools.org/id/palms/sap/keys.php) or classifying vegetation (http://www.lacistemataceae.org/NVC-key.html). Identification keys to a great variety of organisms have also been developed, e.g. for plants (https://gobotany.newenglandwild.org/simple/, http://herbaria.plants.ox.ac.uk/bol/caricaceae/Keys), fungi (http://www.mycokey.com/newMycoKeySite/MycoKeyIdentQuick.html), and animals (http://keyserver.lucidcentral.org/key-server/player.jsp?keyId=68, https://www.rspb.org.uk/discoverandenjoynature/discoverandlearn/birdidentifier/).

With all these facilities, the contribution of non-experts to the accumulation of biological data is increasing, and has already been remarkably successful in providing data on species occurrences and distribution (Bonney et al. 2009).

## Identifying indicator species for conservation planning

Biodiversity research and conservation actions are heavily dependent on the availability of adequate species identifications, whether the aim is to understand species origins, patterns in spatial distribution, or responses of organisms to human impact (Polaszek 2005; Hardisty et al. 2013). However, there is a general lack of taxonomic knowledge, such that even describing all the species is not a realistic goal at present, and providing identification tools for them is even more difficult (Godfray 2002; Wheeler et al. 2004). In poorly known areas, such as Amazonia, one approach to maximize the amount of spatial information from biological surveys is to focus on indicator species (Cajander 1926; Noss 1990; Ellenberg et al. 1992; Ruokolainen et al. 1997, 2007; Margules et al. 2002; Tuomisto et al. 2003a; Salovaara et al. 2004). This lessens the taxonomic identification burden by reducing the number of species that need to be dealt with. Consequently, more sites can be inventoried with the same effort, which facilitates habitat mapping and conservation planning (Noss 1990; Howard et al. 1998; Higgins and Ruokolainen 2004; Ruokolainen et al. 2007).

It has been suggested that ferns are good indicators of environmental conditions, forest types and general floristic patterns (Ruokolainen et al. 1997, 2007). Indeed, ferns are easy to observe and collect, broadly distributed geographically, and several studies have documented that they have specific edaphic affinities (Tuomisto and Poulsen 1996; Tuomisto et al. 1998, 2002, 2003a, 2003b; Tuomisto 2006; Cárdenas et al. 2007; Higgins et al. 2011; Zuquim et al. 2014). Fern occurrence patterns have also been used to test proposed biogeographical barriers (Higgins et al. 2011; Tuomisto et al. 2016) and to map soil properties in areas where soil data were unavailable (Sirén et al. 2013; Zuquim et al. 2014). Therefore, a deeper understanding of fern species distribution patterns can provide relevant information for habitat mapping and conservation planning (Zuquim et al. 2014).

There is an urgent need for more biological data to improve conservation planning in poorly known areas (Feeley and Silman 2011; Feeley 2015), such as Amazonia. Data on correctly identified and georeferenced specimens form a valuable source of information about species distributions in Amazonia, regardless of the degree of formal training of the collector. Since the number of taxonomists is limited, non-specialists can play an important role in recording species occurrences and thereby improving the understanding on species distributions. Unfortunately, there is no comprehensive literature on Amazonian fern species as yet, so identification keys and species descriptions need to be searched for in several scattered publications. Some examples include chapters in a special issue of the journal Rodriguésia (several authors, 2005; e.g., Prado 2005a, 2005b, with 75 species from one central Amazonian locality) and books (Zuquim et al. 2008, with 120 species from another central Amazonian locality; Smith 1995, with 671 species from Venezuela and Cremers 1997, with 182 species from French Guyana). Moreover, new Amazonian fern species and even genera (Moran et al. 2010; Mynssen et al. 2016) are being described, and taxonomic rearrangements necessitate moving some of the already known species to different genera (Kessler and Mickel 2006; Prado 2006; Prado and Moran 2008; Cárdenas et al. 2016). Consequently, printed identification keys may become outdated within a few years.

We developed a free-access online identification tool for Amazonian ferns in order to provide a relatively easy and up-to-date resource for species identification. Our aim was to specifically address the problems mentioned above, and thereby to stimulate the collection of ferns and accumulation of data about them.

## Structuring and implementing the key to Amazonian ferns

**Figure 1.**
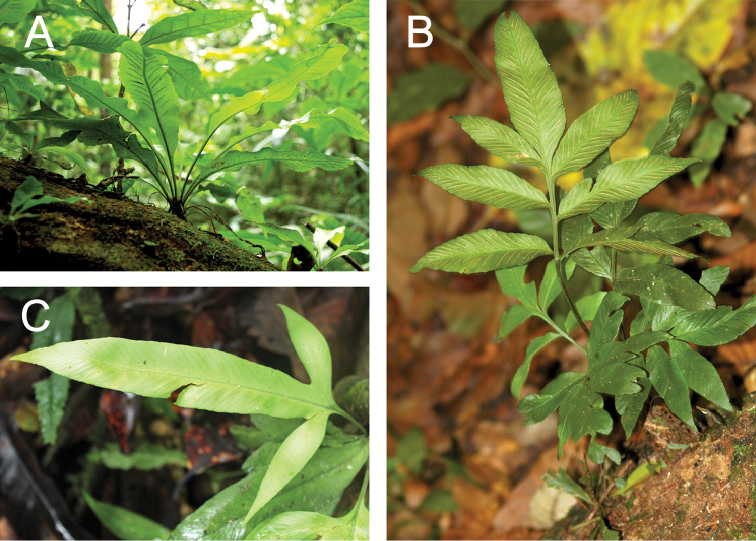
Morphological variation in *Asplenium pearcei* Baker. Three fertile individuals of *Asplenium pearcei* Baker showing different patterns in leaf dissection. **A** entire leaves **B** pinnate leaves and **C** lobed leaves. All states were scored in the features table.

We started the key development by compiling a preliminary list of species and the morphological characters we thought are most useful when identifying them. Preference was given to such external characters that can be observed in the field or in the herbarium with the naked eye. The next step was to decide how the characters and their states are communicated to the user. Rather than programming a key platform from scratch, we decided to use the specialized key design program Lucid v. 3.5 for this purpose. Lucid v. 3.5 treats the taxa as *entities* and morphological characters as *features* that can have two or more *states*. The characters are implemented in Lucid by *scoring*, for each feature, the states that are present in each taxon. For example, the feature “nodes on the petiole” may be scored to have the state “present” for one species (entity) and “absent” for another. A single taxon can have more than one state scored in a feature, so if a species has nodes on some petioles but not all, both states can be scored. Another example from the present key is the feature “leaf architecture” of *Asplenium pearcei* Baker, which is scored for the states “entire”, “lobed” and “pinnate” (Figure 1).

**Figure 2.**
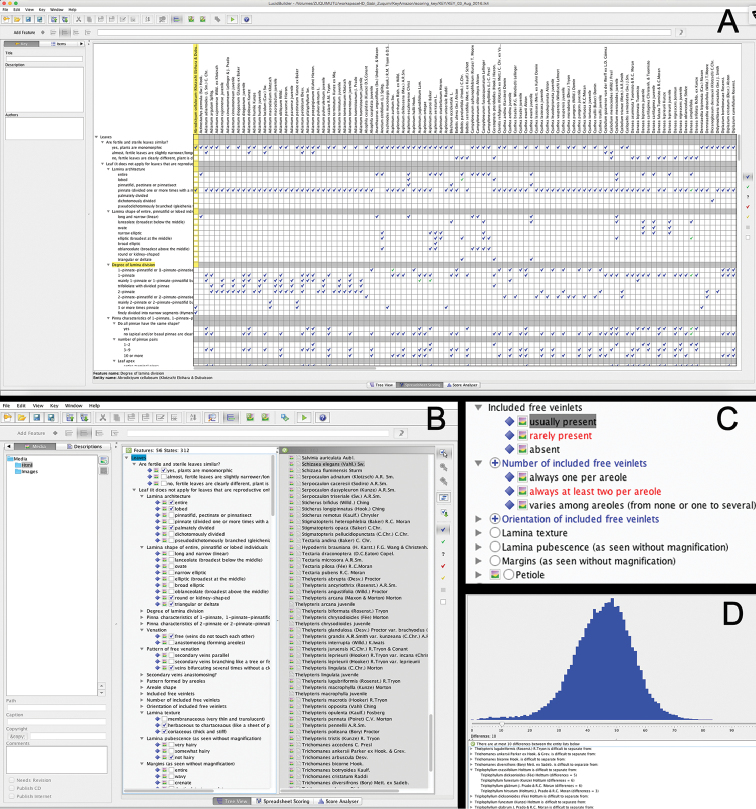
Structure of the key under development in LucidBuilder. Screenshots of the key been developed in Lucid Builder V3.5. **A** spreadsheet scoring table view **B** tree view mode of the scores for a single species **C** an example of features positive relationship: the plus sign associated with “number of included free veinlets” and “orientation of included free veinlets” shows that these features are controlled by the highlighted state (“usually present” under “included free veinlets”). All controlling states for some feature in the key are marked in red **D** frequency plot of the number of differences among pairs of entities and a list of those entities that have at most 10 different scores among them.

Lucid allows scoring features in two different layout modes. The spreadsheet mode shows all features and all entities at the same time, and is useful for mass scoring of features, as well as to get an overview of the data (Figure 2A). The tree view shows all the scores for either one entity or one feature at a time, which is useful when one is adding or checking a single entity or feature (Figure 2B).

There are more than 500 described species in the paraphyletic group known as pteridophytes (ferns and lycophytes) in Brazilian Amazonia (Prado et al. 2015). Our key contains about half of these. We decided to focus on ferns only, because lycophytes are so distinct that they are best treated in a separate key. Within the ferns, we focused on those species that we have learned to know in the field during our long experience in Amazonia. This was necessary both because these are the species we can confidently identify, and because most of the herbarium material used when scoring the features was deposited at TUR. Some species not present at TUR but included in the Uatumã checklist (Zuquim et al. 2009) were also included in the key. All of the species that are included in the key occur in lowland Amazonia. The vast majority can be found in non-inundated (terra firme) forests, but many species typical of white-sand forests and seasonally inundated forests are also included, as well as some species of disturbed areas and a few aquatic species.

The list of taxa in the key is not complete yet, but accommodating more families, genera, and species is straightforward. The features can be applied to any fern species, and more states can be added if the new species are not adequately described by the existing states. For example, at the moment the key contains no species that are hemidimorphic (only a part of the leaf is fertile). When a hemidimorphic species (such as *Anemia*) gets added, a state “leaf divided into a fertile and sterile part” could be added to the feature about the morphology of fertile leaves in relation to sterile ones.

Features that are relevant to a few taxa only were included in separate subkeys. For example, the number of cells in the hairs on the lamina is a diagnostic feature in species of the genus *Triplophyllum* Holttum, but this is a rather difficult character to observe, and in most other genera it is uninformative. Therefore, the feature was only included in the *Triplophyllum* subkey, which is then embedded in the main key. The subkey can be accessed either as link through the main key or independently from the key portal (http://keyserver.lucidcentral.org:8080/sandbox/keys.jsp). More subkeys are under development for other taxa.

Subkeys can also be used more generally to provide modular structure to the key. They can be developed independently for a taxonomic group of interest or for a geographic region and then linked to the main key. If new geographical areas were to be included in the key, a practical option might be to add region and/or biome as one of the features, so the user can subset the key to the geographic area of interest (for example, Amazonia, Panama, Neotropics, Andes, etc.).

Regardless of the identification key format, some morphological features are only meaningful in the presence of a specific state of another feature. For example, “number of pinna pairs” is a relevant feature only among those ferns that have pinnately divided leaves in the first place. A conditional list of features is implemented in Lucid by ascribing logical relationships among characters (or dependencies, following Lucid´s terminology). In other words, Lucid keys can be designed such that the feature “number of pinna pairs” is unavailable (i.e., invisible to the user) until the user scores the state “pinnate” in the feature “leaf architecture”. At that moment, features that are relevant for pinnate leaves automatically appear in the features list. This is called positive dependency (a dependent feature appears when a controlling state is selected). Lucid also allows for negative dependency, which means that the dependent feature is initially available, but disappears from view when a controlling state is selected in another feature that makes it irrelevant. The planning of dependencies is an important part of key design, because when they are well used, they help to keep the features list concise and the appearance of the key more inviting. Our fern key uses positive dependencies but not negative ones. For example, the feature “number of veinlets” is not visible when the key is launched, but it has a positive dependency with two of the states in the feature “included veinlets”, namely “usually present” and “rarely present” (Figure 2C). If the user chooses one of these states, “number of veinlets” automatically appears.

Each species in our key is scored for a higher taxon membership, i.e. genus and family. This provides a quick possibility to subset the key when the user already knows the genus or family. Scoring one of these taxa for the specimen to be identified causes the key to work as a key to species within that taxon only. Taking into account that the key is still missing many species known to occur in Amazonia, we recommend that the user checks from other sources if it seems that none of the species of the key matches the plant needing identification.

**Figure 3.**
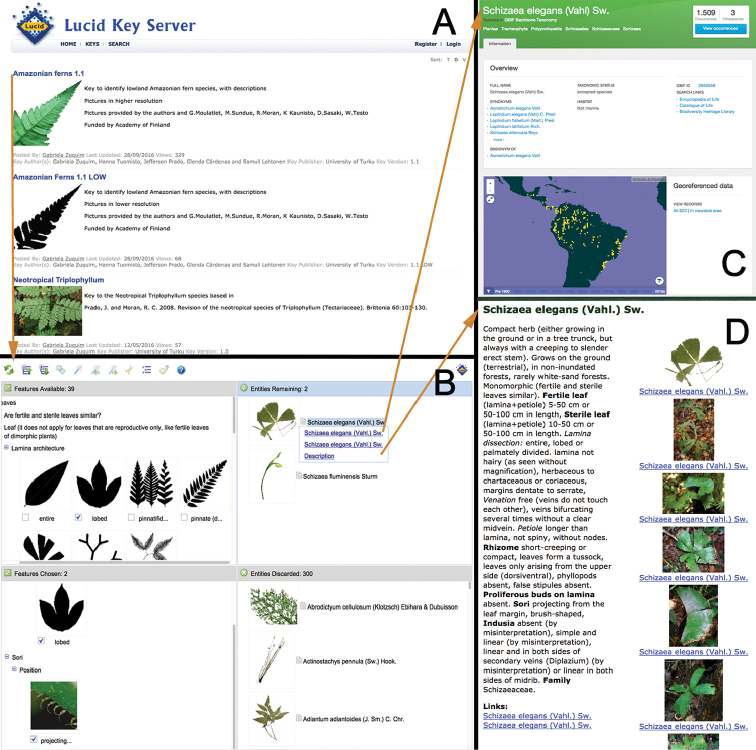
Available contents of the online fern keys. Screenshots of the online key. **A** Entrance page with a list to all available keys **B** an overview of the key after two features have been scored, (C) link to possible identification occurrence map in GBIF **D** description of the entity that possibly matches the plant needing identification.

We used a template to transform the scores of each entity into automatically generated text descriptions (Figure 3D). The descriptions allow the user to view taxon scores in a compact format, and also to access all the pictures that are related to the taxon in the key. In addition, links are provided to external data sources, in particular a map of species records available in the Global Biodiversity Information Facility (GBIF; Figure 3C) and species name status according to The Plant List (http://www.theplantlist.org).

We included more than 5,000 illustrations (photos and drawings) in the key to document the morphological variation within each species and to illustrate the features and their states that were used in the character list. Some of the photos are associated with voucher specimens deposited in TUR, INPA, or SP Herbaria. Species names were updated according to PPG I (2016).

We tried to avoid botanical jargon, but sometimes the precise scientific terms were needed. Then we illustrated them and/or defined them in a glossary that can be accessed in the following link: http://www.utu.fi/en/sites/amazon/publications/Pages/Glossary.aspx

## General aspects of the online key

Lucid identification keys are structured in four tiles containing (a) available features, (b) chosen features, (c) entities remaining, and (d) entities discarded (Figure 3B). Our key includes 302 entities, of which 253 are different species and 59 are separate entries for the juveniles of species that change considerably in appearance during their ontogeny. For example, *Salpichalena volubilis* has three entries, because its small juveniles have entire leaves, intermediate juveniles have once-pinnate leaves, and adult plants have 2-pinnate twining leaves. The list of features contains a total of 54 morphological characters. Of these, 19 are immediately available and the remaining ones have positive dependencies, so they become visible only if they are needed.

Of the 45,451 possible combinations of pairs of taxa, 99.3% pairs were differentiated from each other by more than 10 features (not counting “Family” and “Genus”) (Figure 2D). The pairs of species that no differentiating features were included in the key were *Sticherus bifidus* (Willd.) Ching and *S. longipinnatus* (Hook.) Ching; *Vandenboschia collariata* (Bosch) Ebihara & K. Iwats. and *V. radicans* (Sw.) Copel.; and the juveniles of *Lindsaea bolivarensis* V. Marcano and *L. coarctata* K.U. Kramer.

Since ours is a free-access key, the user can select and score the features in any order, and not all features need to be scored. This represents an important improvement in relation to the usual fern identification keys, which have a single-entry structure. Having to identify a specimen using a pre-determined sequence of features is problematic especially because most keys start with reproductive characters, which are absent in many (if not most) of the individuals one is likely to encounter in the field. The present Amazonian fern key includes features related to both fertile and sterile leaves independently, which makes it possible to identify individuals regardless of its reproductive state. The key can be used to identify fertile fern individuals of any size, but sterile individuals only if they have leaves longer than 10 cm. Although it is possible to identify even smaller juveniles than this, their features are difficult to express, and adding them could make the key confusing.

One possible disadvantage of free-access keys is that the high number of choices can confuse the user (Martellos and Nimis 2015). Moreover, some features can be redundant or uninformative in certain subsets of species. To overcome these problems, Lucid keys present an efficient solution that can be applied via buttons in the toolbar. For example, the “find best” and “next best” buttons calculate and highlight the feature that is most informative to distinguish between the taxa that remain in the list of possible matches. The most informative character of the Amazonian fern key is “indusia”. The most common indusial score reduces the number of potential identifications from 302 to 116 species, which means that one single scored feature reduces the number of possible identification to less than half. Even better, some of the “indusia” scores reduce the number of possible taxa to only one. The next best (i.e., most informative) feature is “rhizome habit”. When scored together, “indusia” and “rhizome habit” reduce the list of remaining entities to 1/6 of the original or fewer. Using these tools, the user can focus on the informative characters and identify a fern individual in a few steps.

A subkey to identify Neotropical *Triplophyllum* (http://keyserver.lucidcentral.org:8080/sandbox/player.jsp?keyId=5&thumbnails=true&gallery=true) based on (Prado and Moran 2008) was embedded in the main key. All the resulting keys are available in the web (http://keyserver.lucidcentral.org:8080/sandbox/keys.jsp). The main page (Figure 3A) contains links to the free-access key to Amazonian species (http://keyserver.lucidcentral.org:8080/sandbox/player.jsp?keyId=19&thumbnails=true&gallery=true&viewer=fancybox), to the same key but with pictures in reduced resolution (for low speed internet connections) and to the *Triplophyllum* subkey. More subkeys are under development. The webpage of the Amazon Team of the University of Turku (http://www.utu.fi/en/sites/amazon/publications/Pages/online-tools.aspx) provides a centralized access to the keys, related glossary, and further information about the project. The keys are also featured in the Ferns of the World – A Digital Herbarium (http://www.fernsoftheworld.com/keys/) and Lucid Central webpages (http://www.lucidcentral.com/en-us/keys173;/searchforakey.aspx). The key runs using the online key player Lucid Key Server with any modern web browser.

## Testing the key: the users’ point of view

Preliminary versions of our key were tested both by pteridologists and by non-specialists, and several changes were done based on their feedback. Workshops were carried out in Finland, USA, Peru, and Brazil (https://amazonkey.wordpress.com/). In Peru and Brazil, university students went to the field to collect ferns and the fresh specimens were identified using the key. The workshop in Finland focused on preserved material. In USA, the workshop was arranged during the international conference “Next Generation Pteridology (2015)” and the focus was on discussing the structure of the free-access key with fern specialists.

Some outcomes of the workshops were: 1) Even though the order in which the features are presented is not relevant in a free-access key, users tended to score the features in the order that they were listed. Therefore, features were re-sorted such that they start from the easiest and most informative ones. 2) Among the material collected in the field, juvenile individuals often remained unidentified or were even misidentified. This was especially the case with species whose habit and/or laminar dissection characters are very different in juveniles and adult plants. For example, juvenile *Lomariopsis*, which have simple entire leaves, were sometimes misidentified as *Asplenium serratum* or *Elaphoglossum*. Therefore, we added juveniles as separate entries that can be identified independently of the adult plants. For example, *Lomariopsis prieuriana* Fée now appears twice in the entities list of the key: one entry refers to the pinnate adult form and the other to entire-leaved juveniles.

## Conclusions

Ferns can be used as indicators of environmental conditions in Amazonia, so mapping fern species distribution can contribute to producing habitat maps, to describing biogeographical patterns and to conservation planning. For all these purposes, a limiting factor is the poor availability of georeferenced and accurately identified species observations. In order to assist in the species identification problem, we have developed a user-friendly free-access key that is available online and summarises some of the existing taxonomic information about Amazonian ferns.

Online keys can be designed such that they require little taxonomic background knowledge, if they focus on intuitive and/or well-explained morphological characters. Our key is almost entirely based on features that are easily observable with the naked eye, and we avoided jargon as much as possible and clarified the terms when needed. Especially the taxa but also the features are richly illustrated, which allows visual comparison between the specimen and the candidate species. We also tried to keep the appearance of the key simple by taking advantage of tools that allow keeping the visible feature list concise and to quickly subset the features and species lists. More species and features can be added to the key any time. Subkeys to the larger and more difficult families and genera can be embedded, similarly to the current *Triplophyllum* subkey. The structure of the key is dynamic and flexible, so in addition of being immediately useful, there is ample room for further development. The next phases will be to expand the key to cover the rest of Amazonian fern flora, and preferably species of adjacent areas as well and to develop more subkeys. We would like to invite colleagues to collaborate on this endeavor.
